# Supplementary material for: SWATH-MS Based Proteomic Profiling of Prostate Cancer Cells Reveals Adaptive Molecular Mechanisms in Response to Anti-Androgen Therapy
Source: Cancers (Basel). 2021 Feb 9;13(4):715. doi: 10.3390/cancers13040715 (PMC7916382; doi:10.3390/cancers13040715)
Supplement: Supplementary file 1 [file cancers-13-00715-s001.zip › cancers-1057912 Supplementary Materials-done.docx]

Supplementary Material: SWATH-MS Based Proteomic Profiling of Prostate Cancer Cells Reveals Adaptive Molecular Mechanisms in Response to Anti-Androgen Therapy

Chamikara Liyanage, Adil Malik, Pevindu Abeysinghe, Judith Clements and Jyotsna Batra


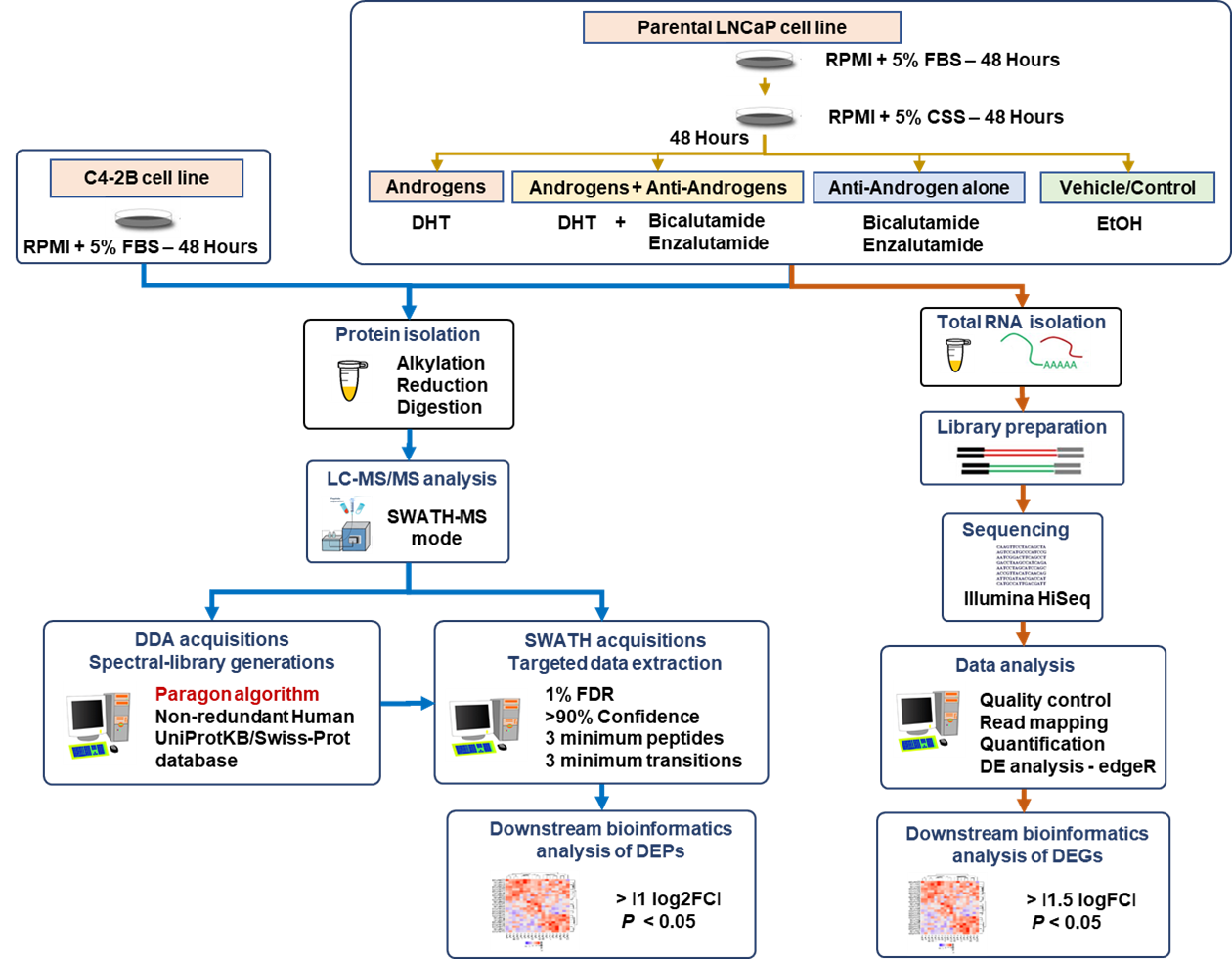


**Figure S1.** Workflow of the proteomic and transcriptomic analysis of androgen and anti-androgen treated of LNCaP cells and C4-2B cells. LNCaP cells were cultured in RPMI-1640 + 5% CSS media supplemented with either DHT and/or BIC/ENZ or EtOH. The C4-2B cell line was cultured in RPMI-1640 + 5% FBS media. Protein isolated from androgen and anti-androgen treated LNCaP and C4-2B cell lines was used for the SWATH-MS/MS analysis for the quantitative comparison of proteomic abundances. RNA isolated from androgen and anti-androgen treated LNCaP cell line was used for the RNAseq analysis for the quantitative comparison of transcriptomic abundances.


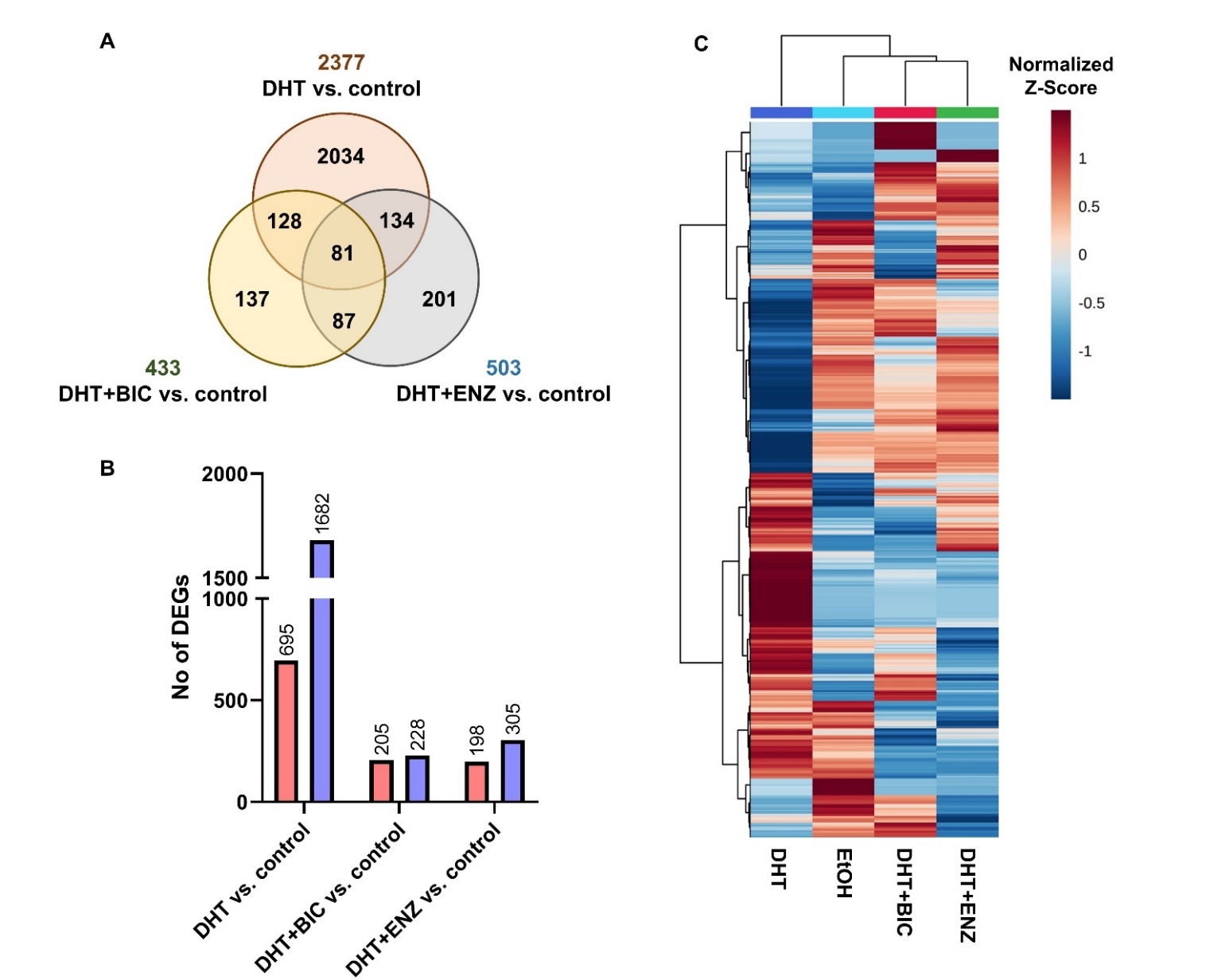


**Figure S2.** Summary of the RNAseq analysis of androgen and anti-androgen treatment of LNCaP cells. (**A**) The number of DEGs overlapped between DHT, DHT+BIC and DHT+ENZ treatments compared to control treatment. (**B**) The bar chart represents the number of upregulated and downregulated DEGs in all treatment comparisons. (**C**) Hierarchical clustering analysis of androgen and antiandrogen treated LNCaP cells. For the Heatmap visualization, normalized Z scores were clustered based on Ward algorithm and row/column orders were set by applying a hierarchical clustering based on Euclidean distance.


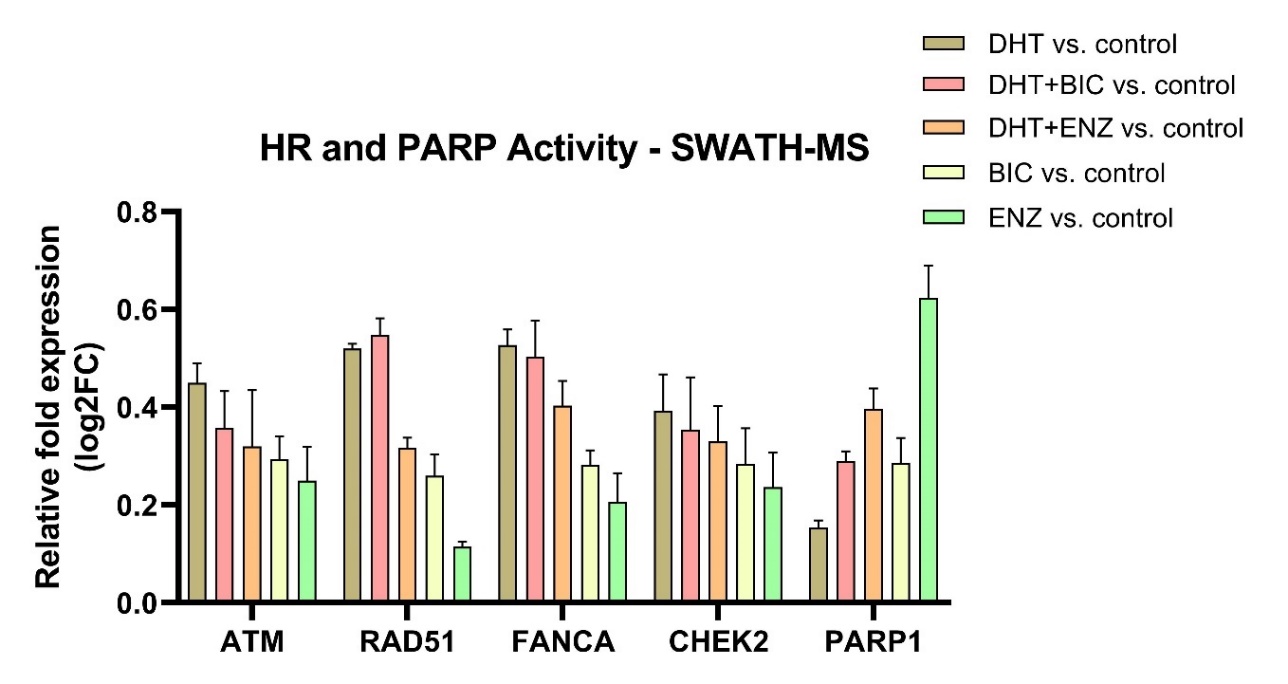


**Figure S3.** Homologous recombination (HR) related protein and PARP protein expression in androgen and anti-androgen treated LNCaP cells. All measurements were taken as log2 fold change expressions in androgen and anti-androgen treatments relative to control (EtOH) treatment. Error bars represent the means ± SD.

| 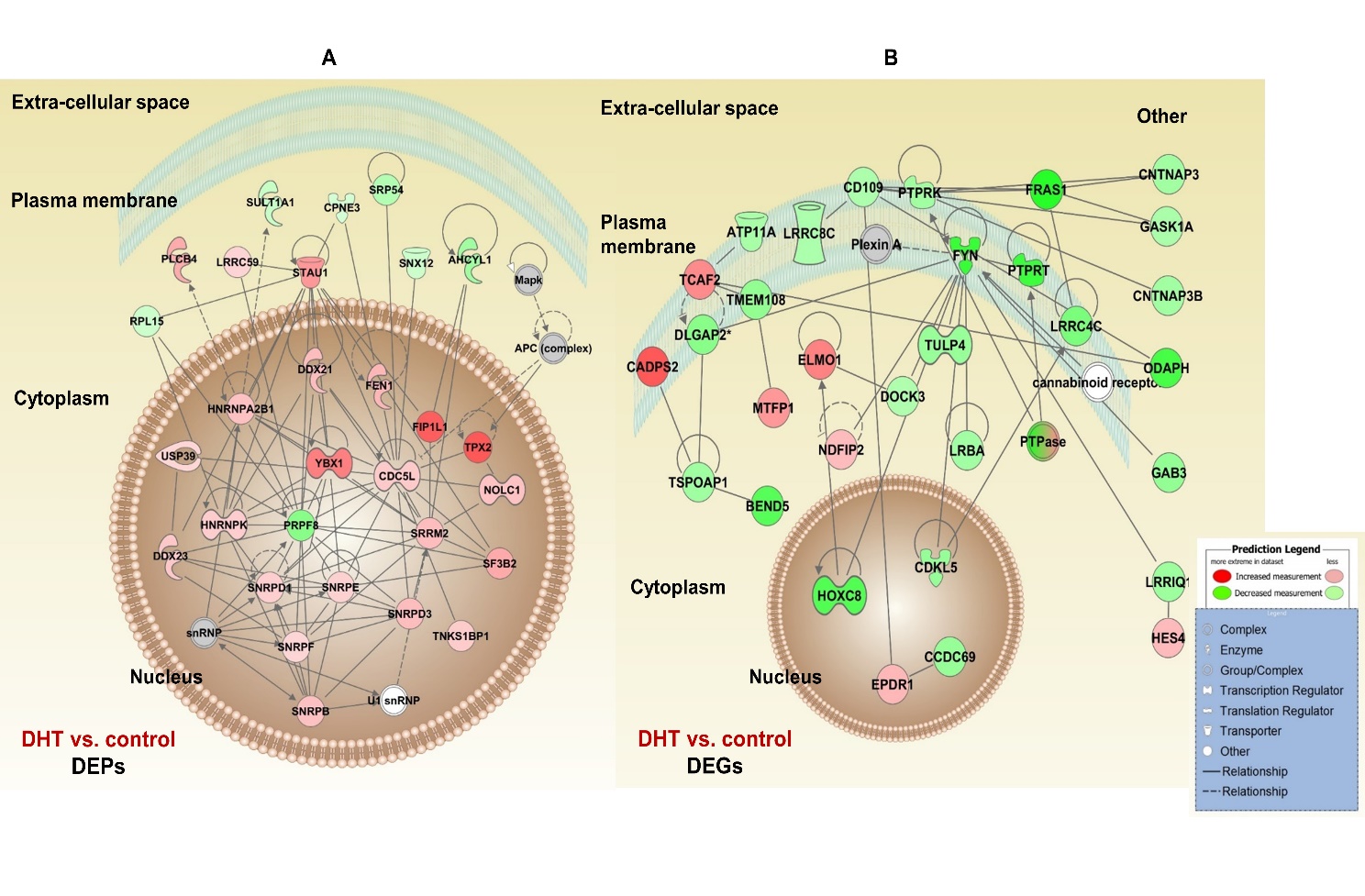 |
| --- |
| 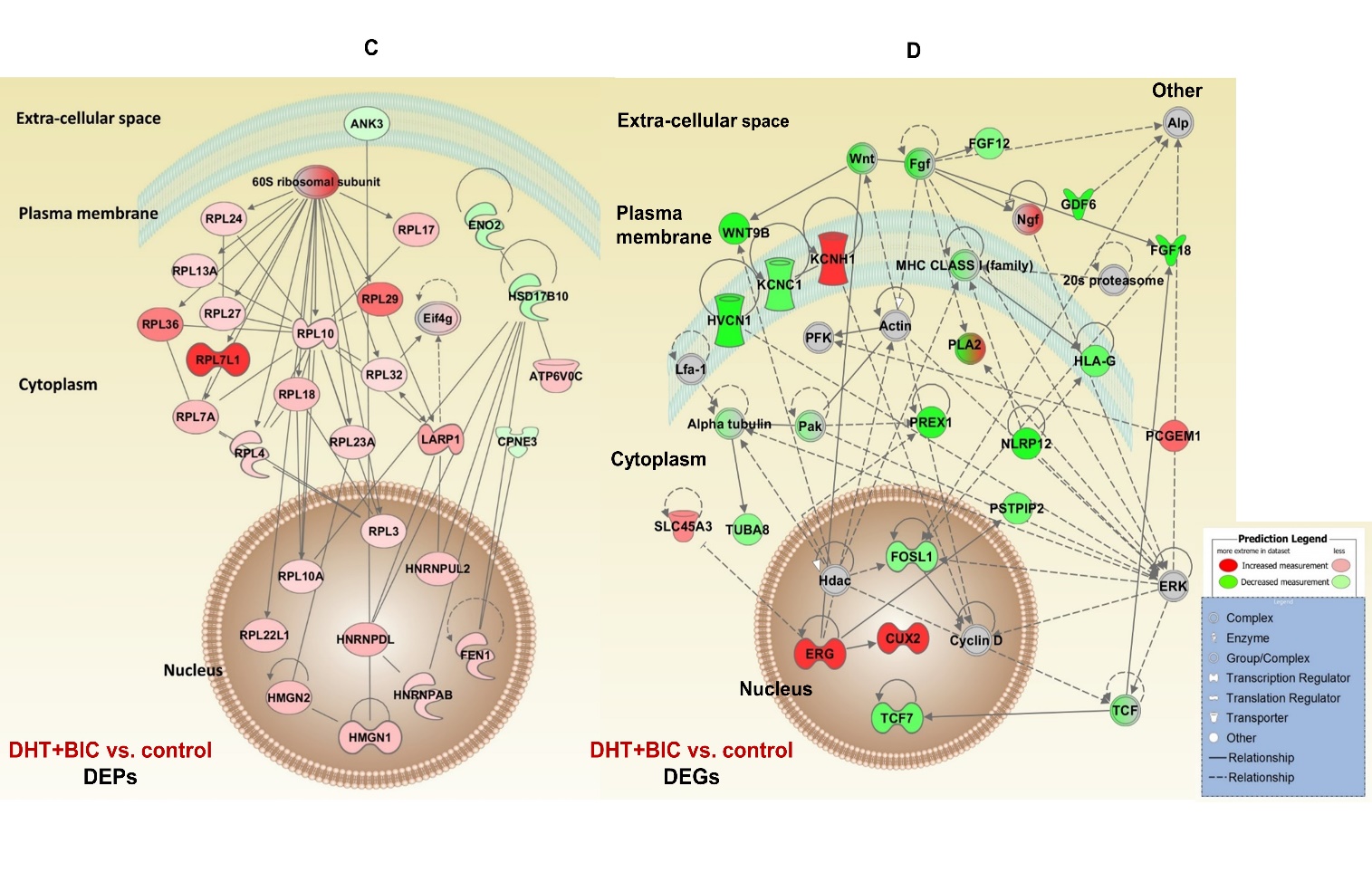 |
| 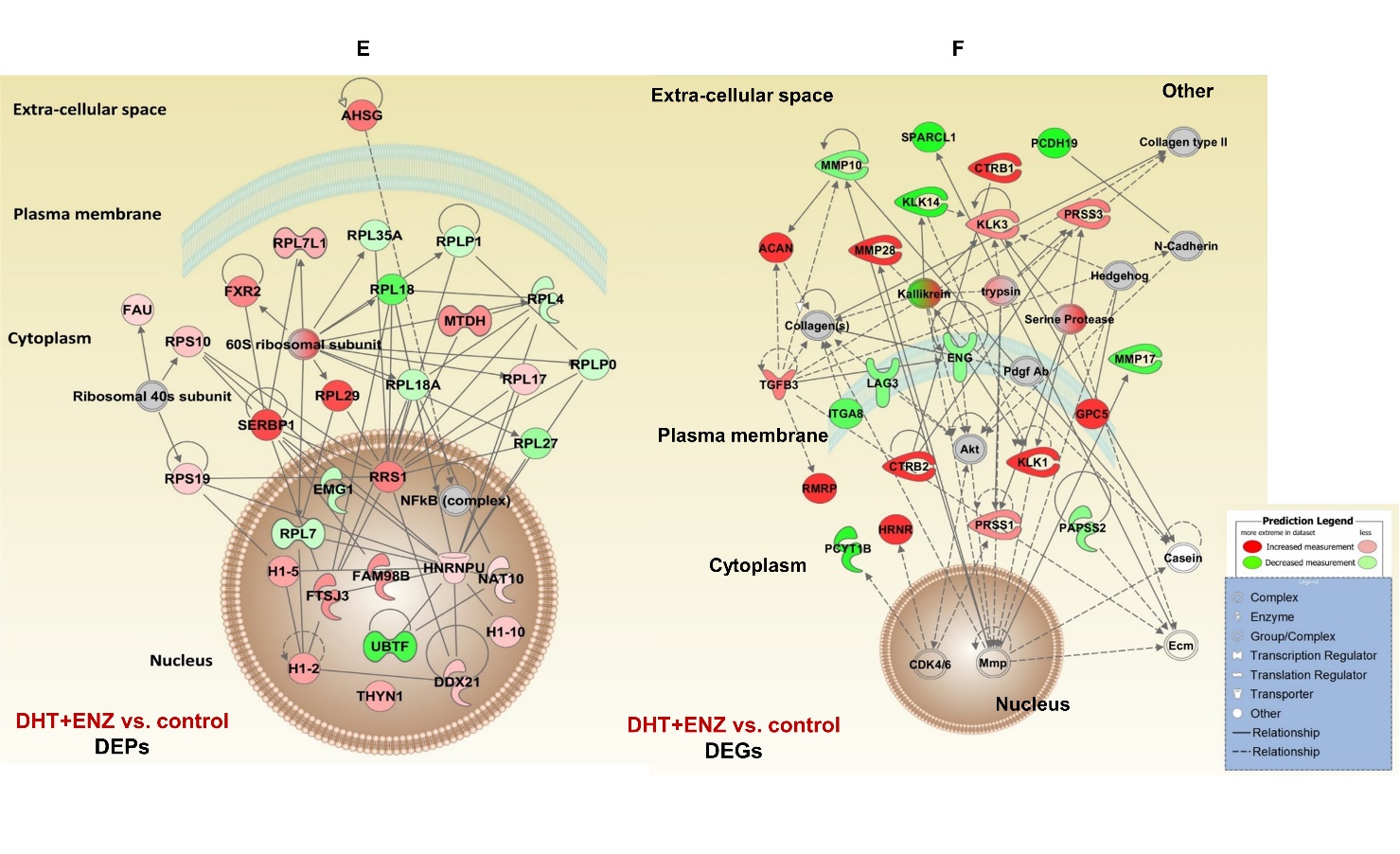 |

**Figure S4.** Top scoring gene networks characterized by androgen and anti-androgen treatments. Protein and gene networks identified by the IPA analyses of (**A**) DHT treatment-DEPs: RNA post-transcriptional modification and cellular organization (**B**) DHT treatment-DEGs: Cellular movement (**C**) DHT+BIC treatment-DEPs: RNA-post transcriptional modifications and protein synthesis (**D**) DHT+BIC treatment-DEGs: Molecular transport (**E**) DHT+ENZ treatment-DEPs: Protein synthesis and RNA damage and repair (**F**) DHT+BIC treatment-DEGs: Post-translational modification. The shapes represent the molecular classes of the proteins. In the figure, red represents upregulation and green, downregulation, and colour intensity represents the relative magnitude of change in protein expression. Direct and indirect interactions are indicated by solid, and dashed lines, respectively. Only DEPs/DEGs with *p* < 0.05 are showed in the networks.


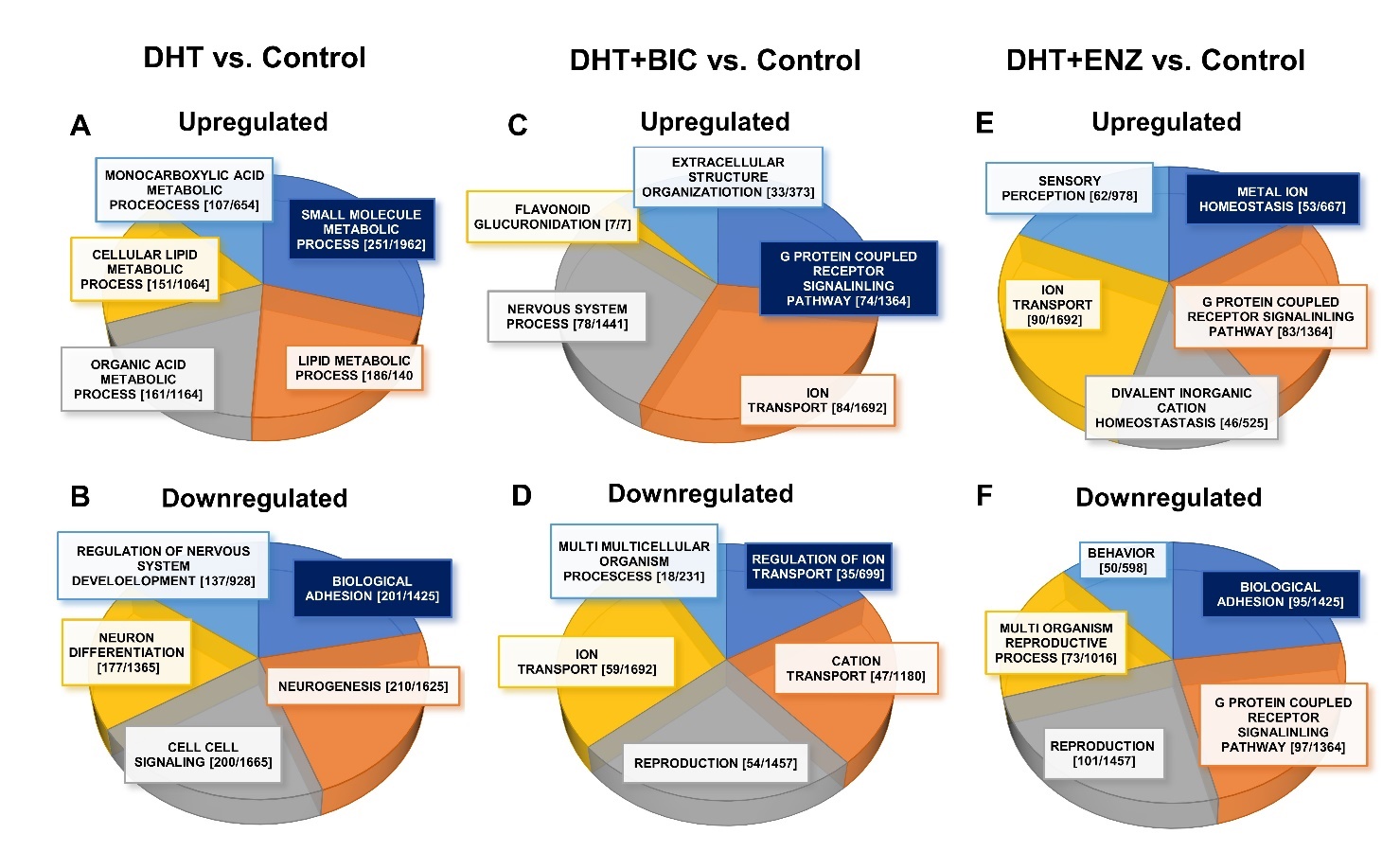


**Figure S5.** GO-biological processes overrepresented by the genes up-/down-regulated in response to androgen and anti-androgen treatment. The pie charts represent the GO-biological processes significantly dysregulated (FDR/q < 0.05) in DHT treatment: (**A**) upregulated (**B**) downregulated; DHT + BIC treatment: (**C**) upregulated (**D**) downregulated; and DHT + ENZ treatment: (**D**) upregulated (**E**) downregulated, compared to control treatment. Highlighted (Blue) biological process represent the most significantly dysregulated GO-biological process. The size of each section represents the associated number of genes associated for each GO biological process. Each GO-biological process is given with [the number of DEGs (k)/ number of molecules GO-BP gene set (K)].

| 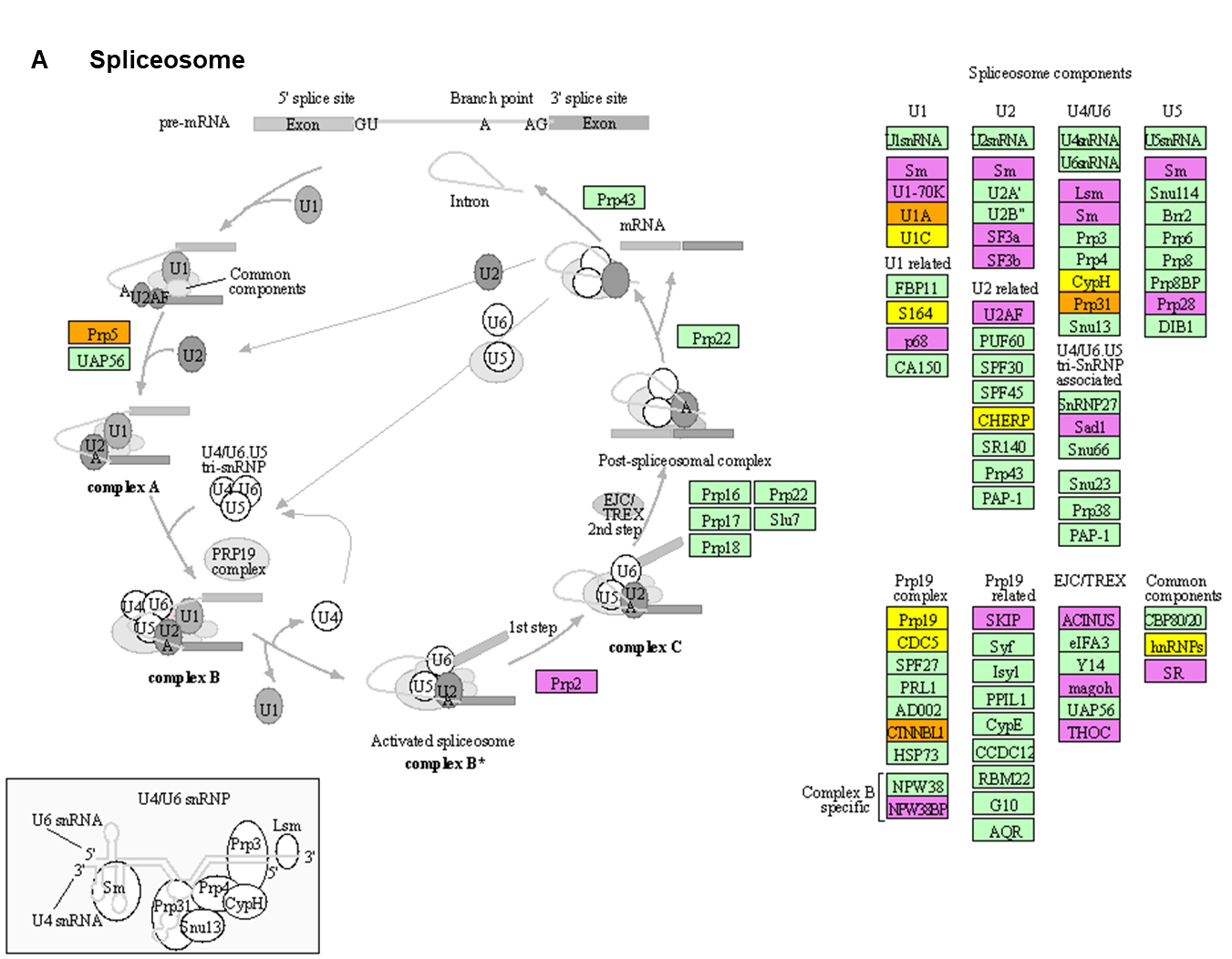 |
| --- |
| 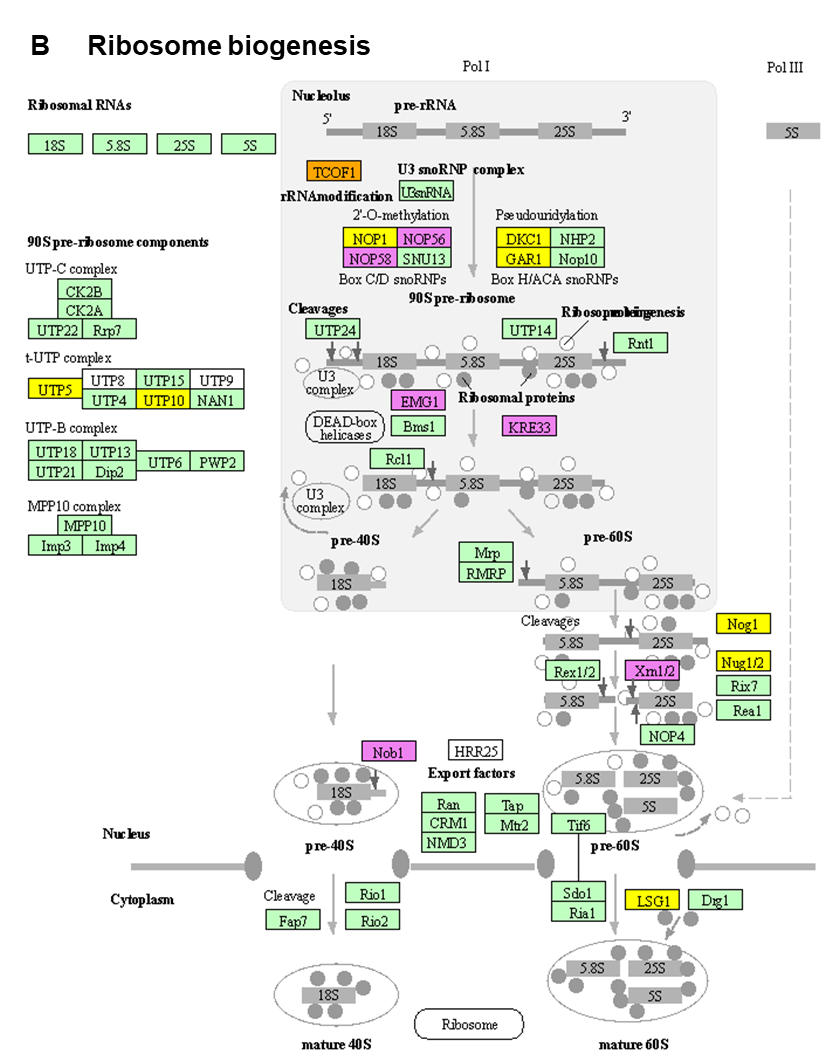 |
| 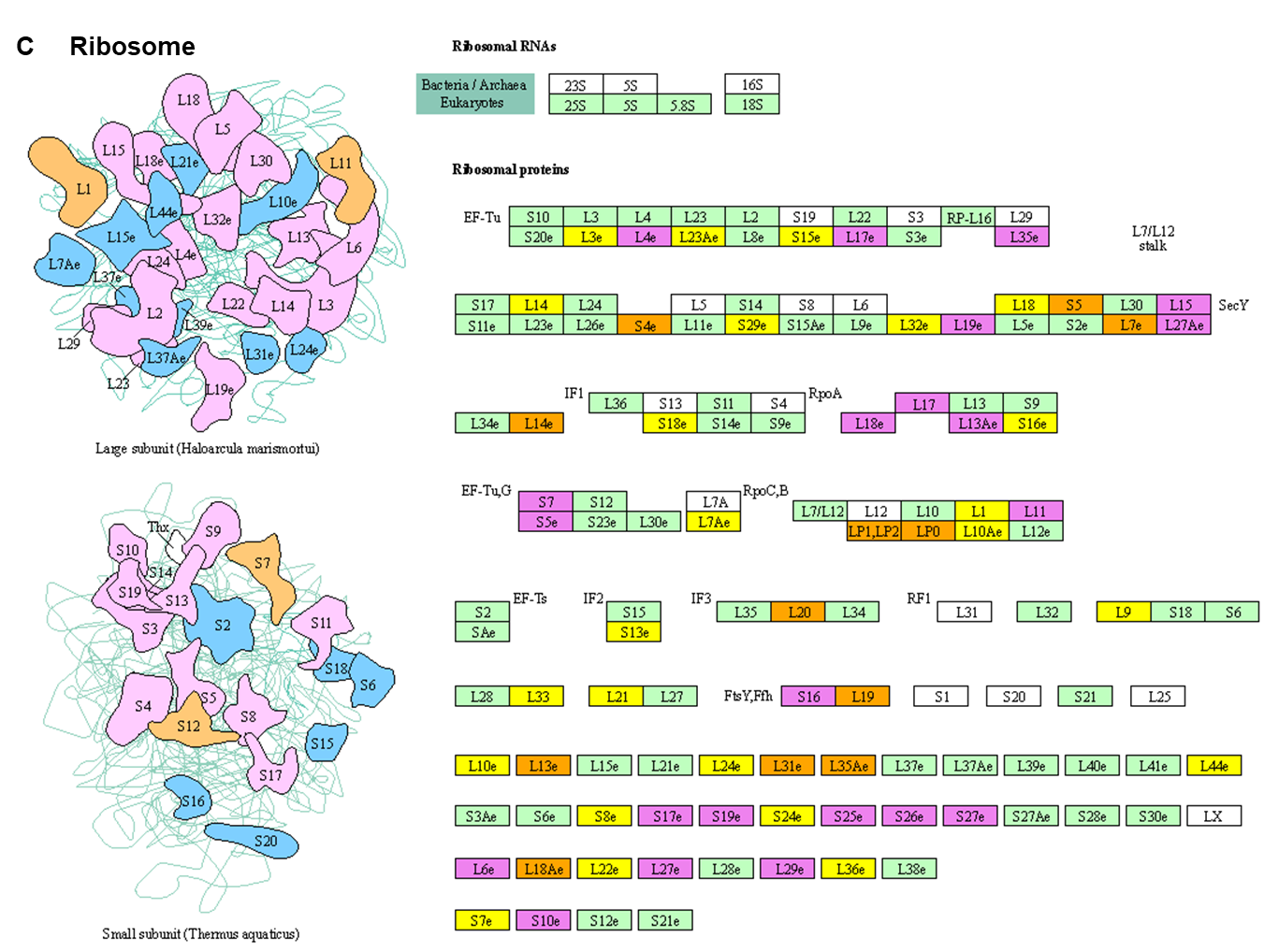 |
| 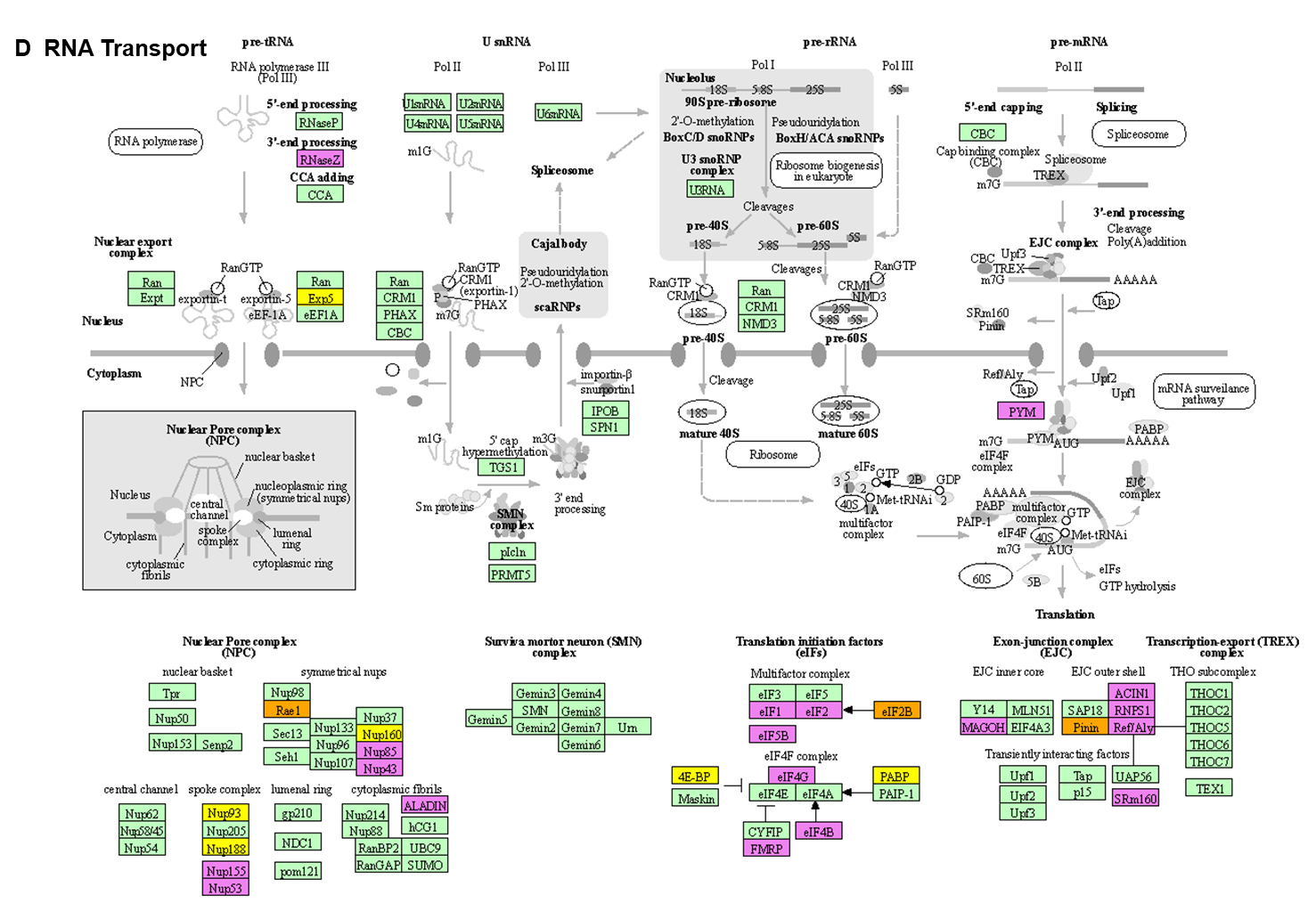 |
| 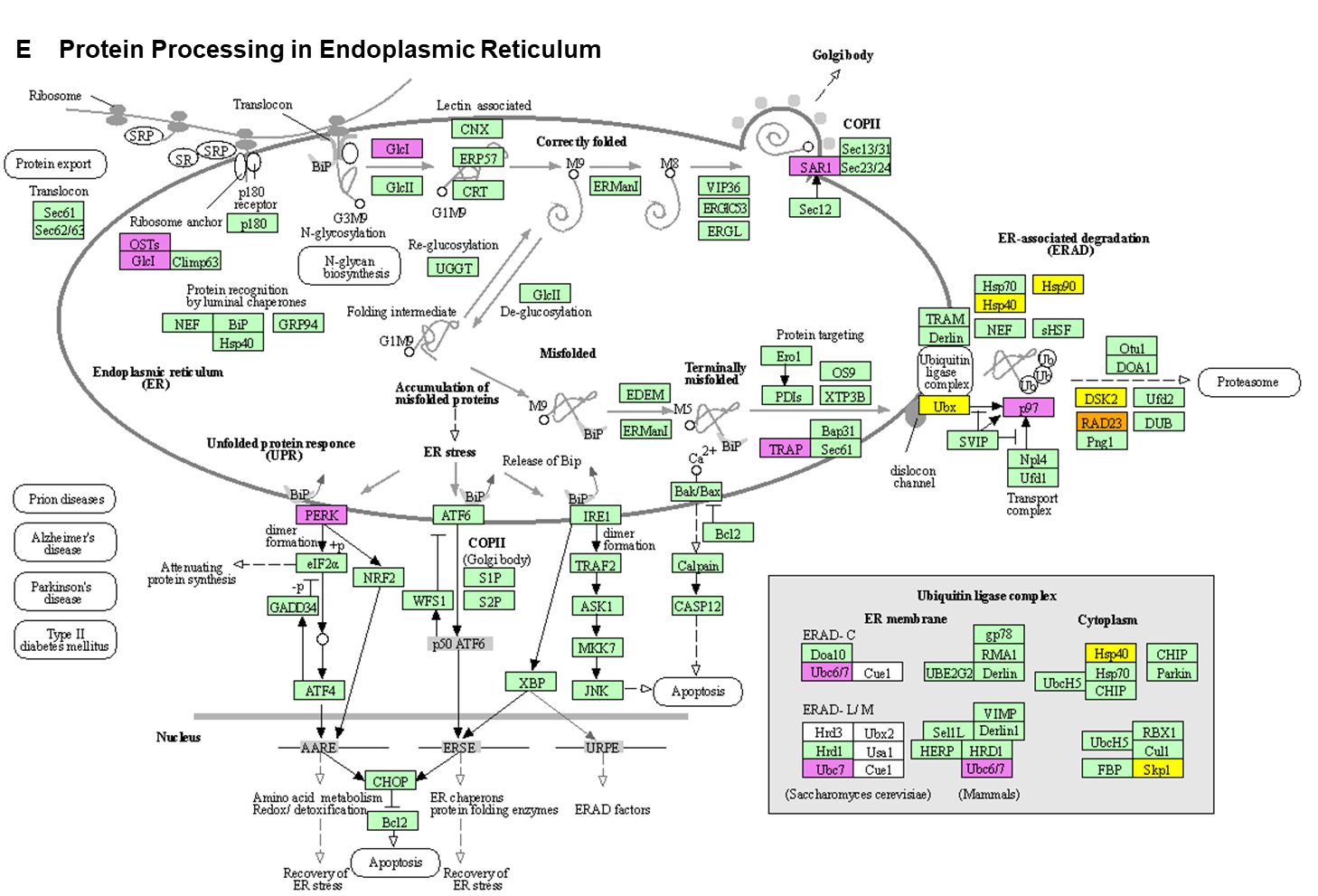 |
| 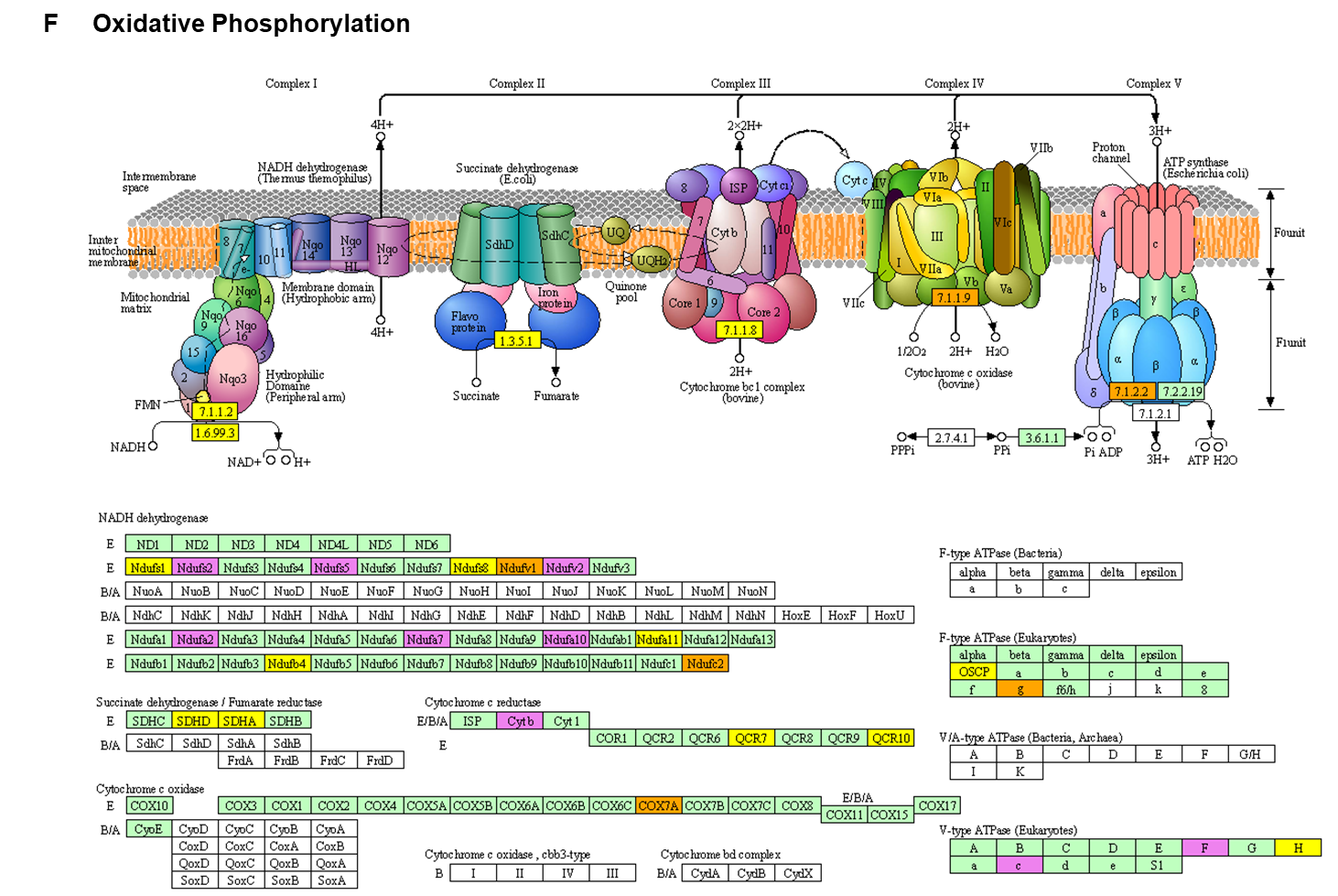 |
| 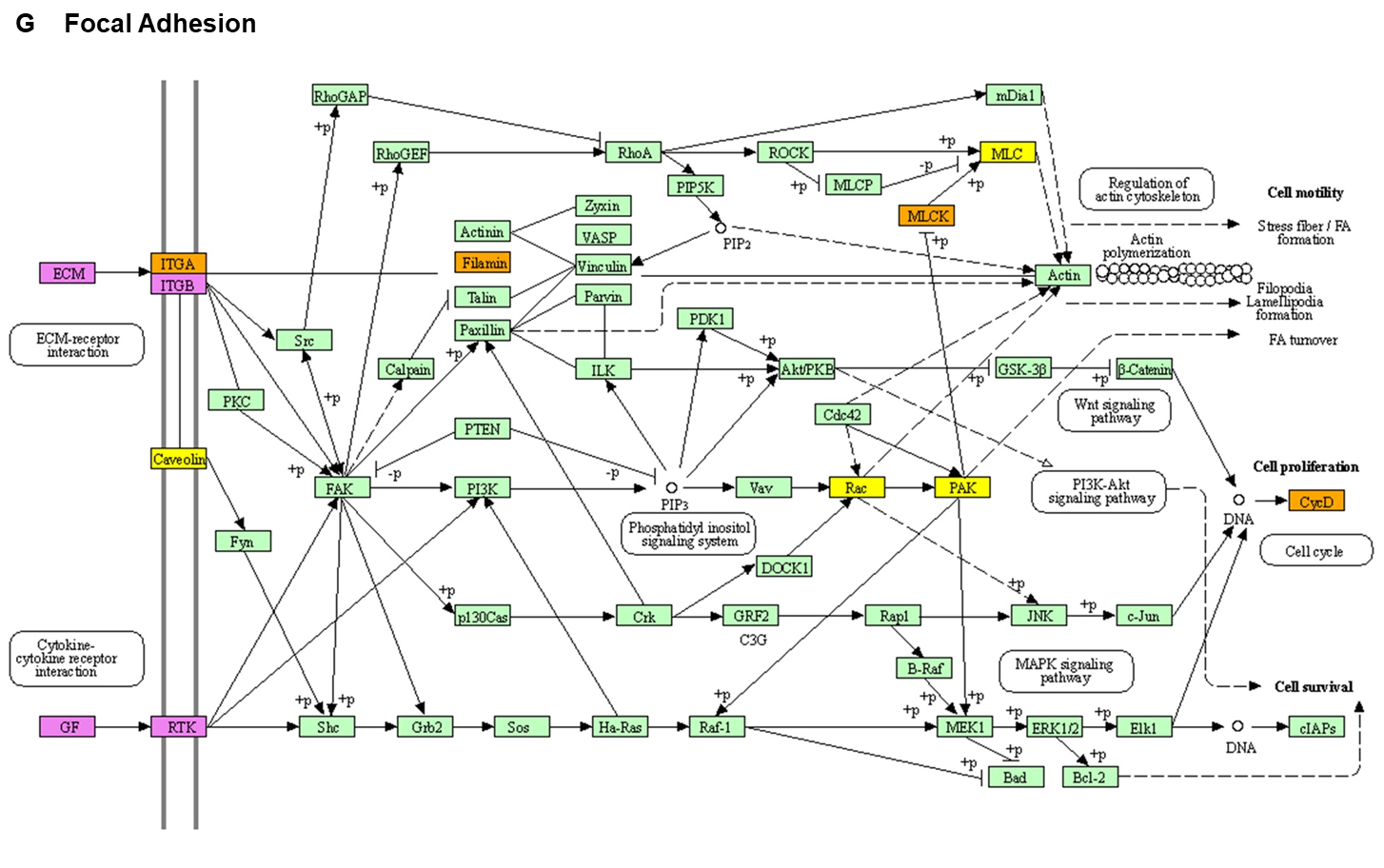 |
| 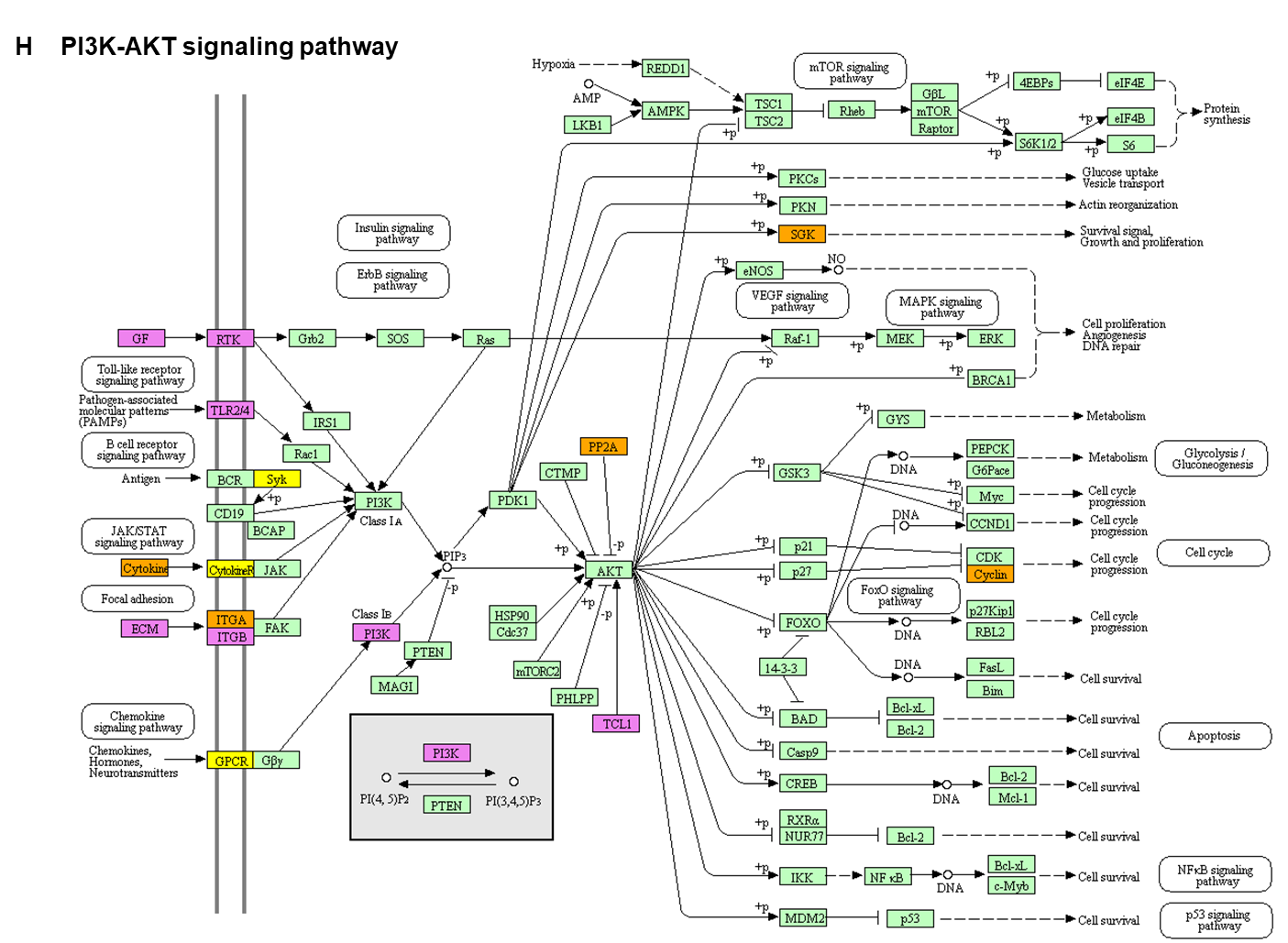 |
| 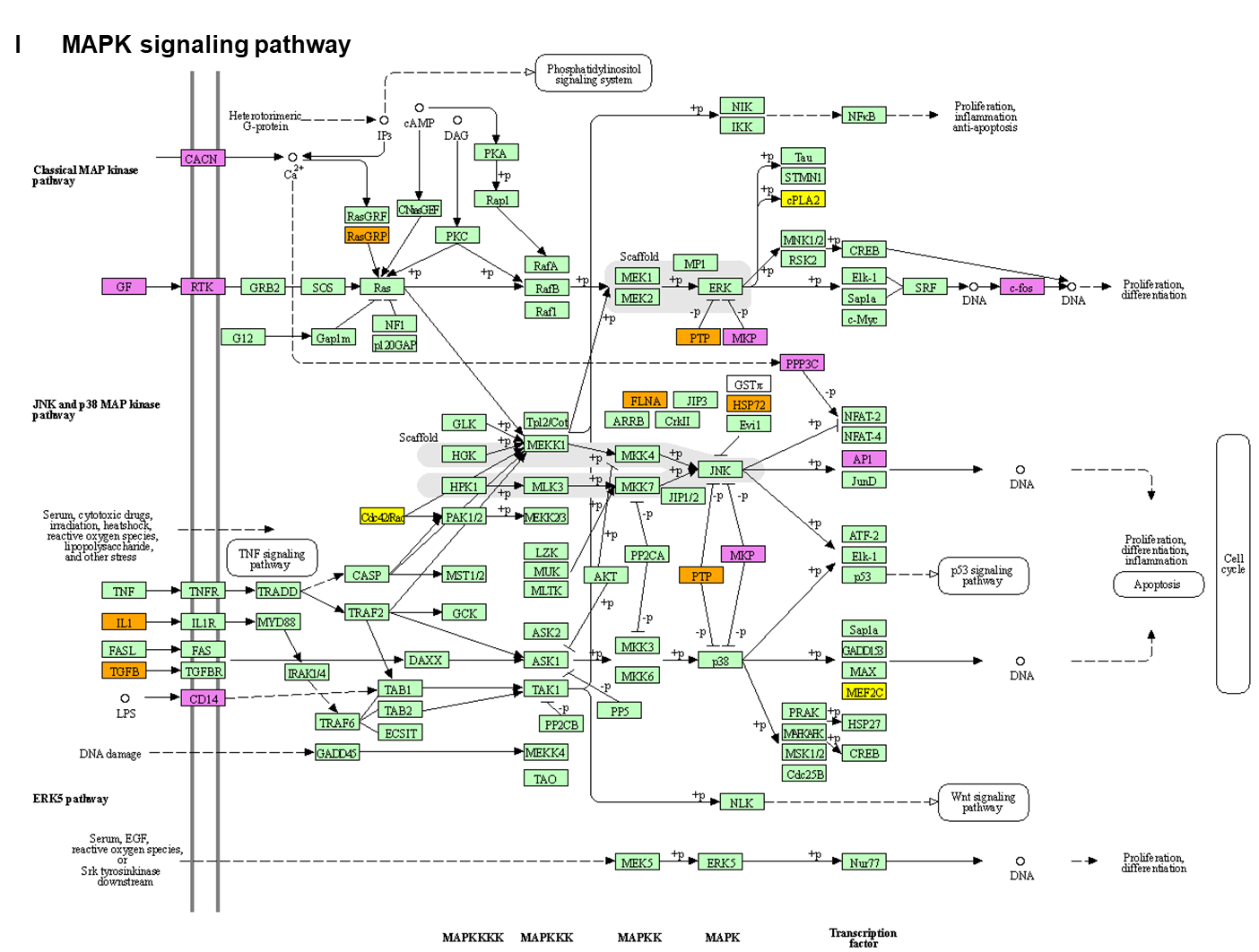 |
| 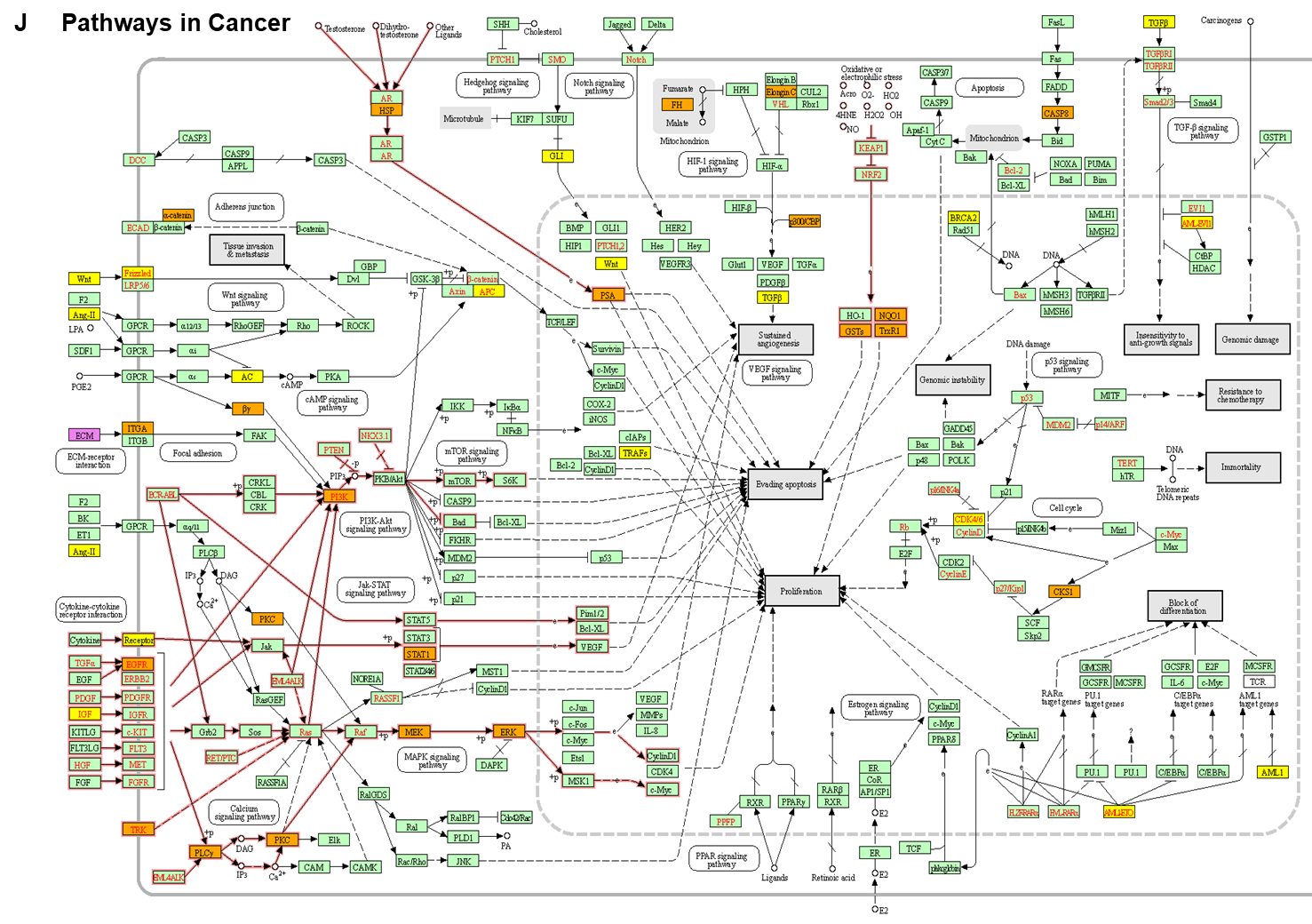 |
| 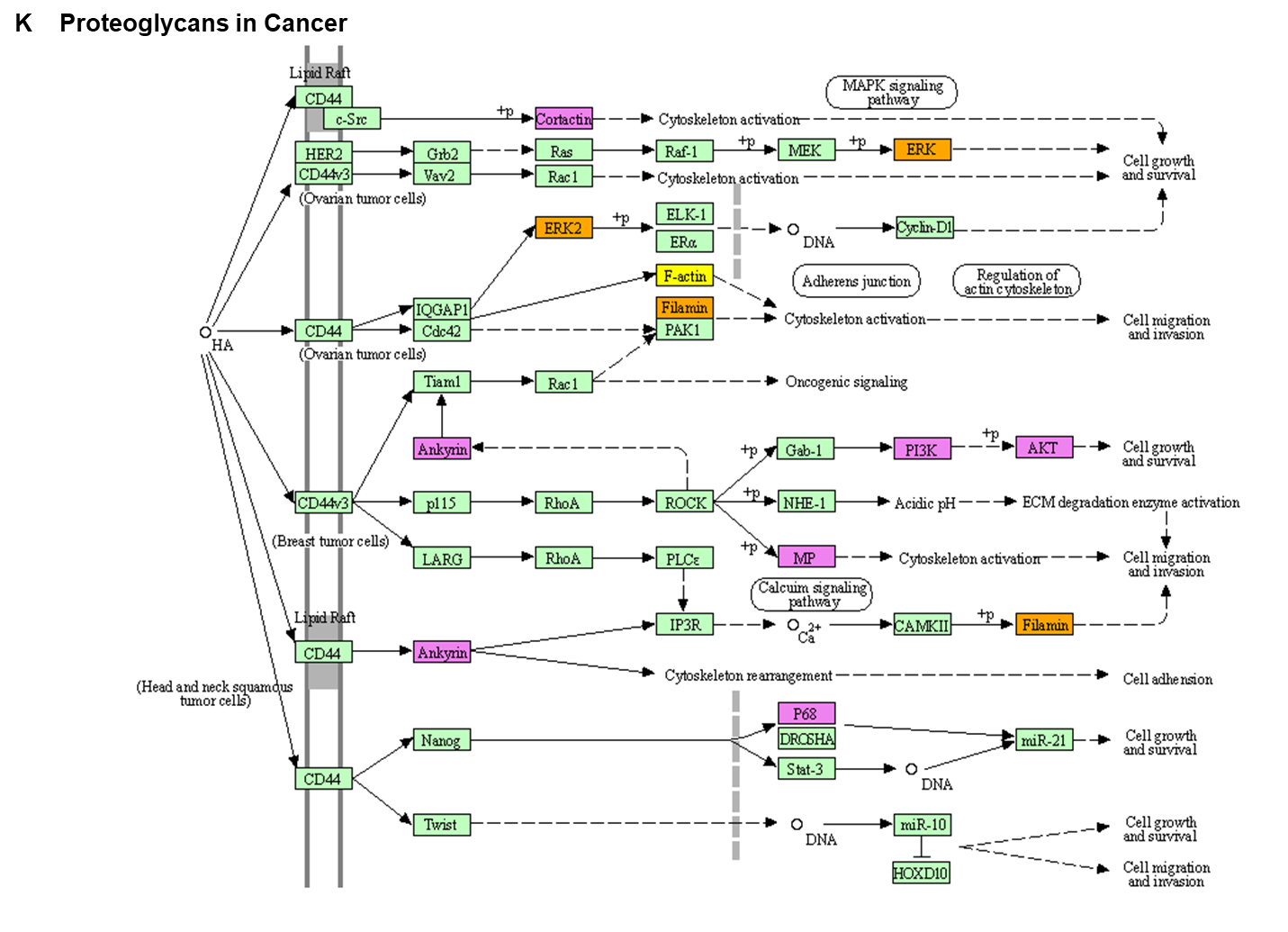 |

**Figure 6.** KEGG pathways differentially regulated during anti-androgen treatment**.** Colored components demonstrate the DEP/DEGs identified in (**A**) spliceosome, (**B**) ribosome biogenesis, (**C**) ribosome, (**D**) RNA transport, (**E**) protein processing in endoplasmic reticulum, (**F**) oxidative phosphorylation, (**G**) focal adhesion, (**H**) PI3K/AKT signaling pathway, (**I**) MAPK signaling pathway, (**J**) pathways in cancer, (**K**) proteoglycans in cancer. Orange: DEP/DEGs identified in DHT+BIC vs. Control, Yellow: DEP/DEGs identified in DHT+ENZ vs. Control, Violet: DEP/DEGs shared between in DHT+BIC and DHT+ENZ vs. Control.

**Table S1** (separate Excel file “Tables S1-S4.xlsx”): Dysregulated molecular networks in response to androgen and anti-androgen treatment.

**Table S2** (separate Excel file “Tables S1-S4.xlsx”): GO-Biological Processes affected by androgen and anti-androgen treatment.

**Table S3** (separate Excel file “Tables S1-S4.xlsx”): Dysregulated upstream regulators in response to androgen and anti-androgen treatment.

**Table S4** (separate Excel file “Tables S1-S4.xlsx”): Top differentially regulated KEGG pathways and associated proteins/genes in LNCaP cells treated with anti-androgens and C4-2B cells.
